# Labonté Identifies Key Issues for Health Promoters in the New World Order: Comment on "Health Promotion in an Age of Normative Equity and Rampant Inequality"

**DOI:** 10.15171/ijhpm.2016.144

**Published:** 2016-11-02

**Authors:** Dennis Raphael

**Affiliations:** School of Health Policy and Management, York University, Toronto, ON, Canada.

**Keywords:** Health Promotion, Sustainable Development Goals (SDGs), Economic Globalization

## Abstract

For over 35 years Ronald Labonté has been critically analyzing the state of health promotion in Canada and the world. In 1981, he identified the shortcomings of the groundbreaking Lalonde Report by warning of the seductive appeal of so-called lifestyle approaches to health. Since then, he has left a trail of critical work identifying the barriers to — and opportunities for —health promotion work. More recently, he has shown how the rise of economic globalization and acceptance of neo-liberal ideology has come to threaten the health of those in both developed and developing nations. In his recent commentary, Labonté shows how the United Nations’ 2015 Sustainable Development Goals (SDGs) can offer a new direction for health promoters in these difficult times.

*“And I always thought: the very simplest words Must be enough. When I say what things are like Everyone‘s heart must be torn to shreds.*

*That you‘ll go down if you don‘t stand up for yourself. Surely you see that.”* Bertolt Brecht^1^

In his commentary, “*Health Promotion in an Age of Normative Equity and Rampant Inequality,”* Ronald Labonté not only br**i**ngs together, with unique insight, recent developments in a wide range of areas not normally conceived of constituting health promotion, he also identifies a unique opportunity for health promoters to move the health promotion agenda forward.^2^

Labonté notes the contrast between the increasing concern with equity in numerous international and national documents and statements and the actual growing inequities between and within nations. He goes on to document the extent of these growing inequities in rather breathtaking terms. This is certainly a situation that many health promoters can identify with. In Canada for instance, there is a plethora of governmental and agency reports about the importance of addressing the quality and equitable distribution of the social determinants of health, yet at the same time precious little public policy that actually does so.^3^

The main contribution of Labonté’s commentary is to bring to the attention of health promoters the potential of the United Nations (UN) Sustainable Development Goals (SDGs) for responding to this crisis of inequality. He points out 193 nations have signed off on the SDG’s such that all world leaders are committed to working towards these goals. While we may question their sincerity, these world leaders will have to account for progress on these goals. This provides an opportunity for demanding accountability in meeting these goals.

The importance of these SDG’s is that while they originate with the UN rather than the World Health Organization (WHO), they constitute an agenda that is hard to differentiate from the traditional health promotion agenda as articulated in numerous WHO declarations and statements.^4^ There are 17 goals with 169 targets. In his commentary, Labonté identifies a short list of six goals all of which will resonate with those working to promote health. The short list includes:

Goal 1: End poverty in all its forms;
Goal 2: End hunger, achieve food security and improved nutrition and promote sustainable agriculture;
Goal 3: Ensure healthy lives and promote well-being for all at all ages;
Goal 4: Ensure inclusive and equitable quality education and promote lifelong learning opportunities for all;
Goal 6: Ensure availability and sustainable management of water and sanitation for all;
Goal 10: Reduce inequality within and among countries; and
Goal 12: Ensure sustainable consumption and production patterns.

Labonté then presents an even shorter list of three goals. These are:

1. Ensure quality education for women and girls (not to ignore men and boys, but emphasizing women and girls can rapidly advance gender empowerment, one of the best known means to improve health equity);
2. Reduce inequality (which in itself should eliminate poverty); and
3. Consume and produce sustainably (which requires more equitable global patterns alongside aggregate global reductions, underpinning all the environmental SDGs).

He then identifies the means by which this can come about. These actions have the potential to engage a wide range of civil society actors in moving forward a progressive agenda. Indeed, they are consistent with recommendations from the health promotion literature on how to develop health promoting public policy to promote health equity.^5^ These activities include:

- Increase the share of economic wealth going to labour (over capital).
- Increase progressive taxation, income redistribution and subsidization of public services and goods.
- Regulate the market for level playing field that is just and environmentally sustainable.

Importantly, Labonté goes on to show how this might come about by increasing labour’s bargaining power, forcing governments to institute greater post market redistribution, and regulating for a more level playing field in the distribution of economic resources.

Labonté identifies distractions to our health promotion efforts. These include the long-standing risk of *lifestyle drift* by which governments ignore the structural determinants of health in favor of attributing adverse health outcomes to so-called individual lifestyle choices.^6^ The second is the development community’s newfound infatuation with the term *resilience* by which attention is directed not to the structural causes of social and health inequalities but rather to the ability of individuals and communities to withstand these adverse situations. The final distraction is what he terms an obsession with the term *innovation.* This implies that what is already known about promoting health should somehow be secondary to hypothetical innovative health technologies that should somehow be able to deal with all the problems identified with promoting health.

I have only scratched the surface of the many insights and gems contained in this commentary. Many of these insights will be unfamiliar to health promoters, even those who have been working in the area of healthy public policy. Not only are the days of promoting healthy behaviors long gone, but also the simple naïveté that advocating and calling for “healthy public policy” will lead policy-makers to implement these.

We are now living in a period where the balance between corporate power and wealth and the health needs of citizens is profoundly out of whack. In this commentary, Labonté identifies a means by which this imbalance can begin to be addressed: *Require governments that have signed off on the SDGs to actually do something about achieving them.* In the process, we can begin to address the structures and processes that create these inequalities in all their forms.

This task may be daunting, but as Labonté argues: “not doing so simply guarantees our failure.”

Labonté’s commentary should be required reading for all those concerned with the health of societies, communities, and individuals. And this should especially be the case for those who go by the name of “health promoters.”

## Ethical issues

Not applicable.

## Competing interests

Author declares that he has no competing interests.

## Author’s contribution

DR is the single author of the paper.
